# Gender-Specific Risk Factors for the Development of Retinal Changes in Children with Type 1 Diabetes

**DOI:** 10.3390/jpm11060588

**Published:** 2021-06-21

**Authors:** Marta Wysocka-Mincewicz, Joanna Gołębiewska, Marta Baszyńska-Wilk, Andrzej Olechowski

**Affiliations:** 1 Clinic of Endocrinology and Diabetology, Children’s Memorial Health Institute, Al. Dzieci Polskich 20, 04-730 Warsaw, Poland; mbasz@wp.pl; 2 Faculty of Medicine, Lazarski University, 02-662 Warsaw, Poland; joanna.golebiewska@wp.pl; 3 Ophthalmology Department, South TEES Hospitals NHS Foundation Trust the James Cook University Hospital, Marton Road, Middlesbrough S4 3BW, UK; olechowski@gmail.com

**Keywords:** type 1 diabetes, children, diabetic retinopathy, diabetes complications, risk factors

## Abstract

The aim of the study was to determine gender-specific risk factor sets which could influence optical coherence tomography (OCT) results in children with type 1 diabetes (T1D). Material and Methods: 175 children with T1D without symptoms of diabetic retinopathy were enrolled, but 330 eyes were used for the final analysis (168 children, mean age 12.81 ± 3.63 years, diabetes duration 4.59 ± 3.71 years). The multivariate regression models for retinal thickness (foveal FT, and parafoveal PFT) and vascular densities (superficial and deep) were carried out separately for both genders using all metabolic and demographic parameters. Results: In the statistically significant multiple regression models for all analyzed OCT parameters for both genders, pH at the onset of diabetes were in existence, as well as for retinal thickness current HbA1c. Duration of continuous insulin infusion (CSII) was an important factor in all parameters, except PFT. For the girls, the most significant factors were daily insulin dose, uric acid, and triglycerides, but for the boys, it was serum creatinine, systolic pressure, and free thyroxine level. Conclusions: We detected significant risk factors set for development of OCT parameters changes, and they were not identical for both genders. Current metabolic control, diabetic ketoacidosis at the disease onset, serum creatinine and longer use of CSII are the most important factors for retinal thickness and vessel densities in both genders in children with type 1 diabetes. For the girls, elements of metabolic syndrome (uric acid and triglycerides) and parameters of insulin amount were more pronounced.

## 1. Introduction

The incidence of type 1 diabetes is still rising and more frequently concerns younger children [1], and even if our treatment methods are significantly improved, the longer diabetes duration has burden impact. Diabetes is a major cause of cardiovascular disease, chronic kidney disease, blindness, and loss of limbs due to amputation in adulthood. There is evidence of clinically significant sex differences in the aetiology, epidemiology, management, and prognosis of diabetes. For many years, diabetologists have been trying to improve and individualize targets, treatment methods and approach to each child and their family, knowing how every year of care has an impact on the future lives of these growing organisms. However, most studies concerning patients with T2D—as confirmed in the meta-analysis by Huxley et al.—have found that women with T1D are more prone to vascular events than men [2], despite the fact that normally women are at lower risk of coronary heart disease. Existing differences in the prevalence of risk factors such as waist-to-hip ratio, obesity, insulin resistance, dyslipidemia, hypertension, coagulation factors, endothelial dysfunction and systemic inflammation are often greater in women [3,4,5,6]. Results of many studies on sex differences in risk factor screening, control and drug interventions in diabetes show that the burden is mainly increased in women, or there are no meaningful conclusions [7]. Nowadays, there is low risk of developing DR in children. However, in the literature there are cases of adolescents with diabetic macular oedema or even proliferative diabetic retinopathy (PDR) [8,9,10,11]. Patomechanisms of retinal vasculature damage are not fully explained; nevertheless micro and macroangiopathy are the likely reasons. OCT and OCTA now give new tools for non-invasive, measurable, repeatable diagnostic of retina and ophthalmic vascularity [12]. In our previous studies in numerous groups of children and adolescents with T1D with no signs of retinopathy, we revealed associations with OCT and OCTA results with many metabolic and demographic parameters [13,14]. We hypothesize that early detection of suboptimal risk factors allow for the planning of subsequent interventions—either pharmacological or lifestyle—which significantly improve clinical outcomes. The aim of our study was to check whether there are any groups of factors which are especially important for girls or boys, and what therefore could be the target for personalized therapy. Based on such an assumption, we performed an analysis of all collected demographic and metabolic data and prepared multiple regression models for OCT and OCTA parameters in a group of T1D children without retinopathy.

## 2. Material and Methods

This prospective, observational study enrolled 175 Caucasian children with a T1D diagnosis based on ISPAD criteria and with insulin treatment (not in full remission). Exclusion criteria were a history of prematurity, other concomitant retinal pathologies, such as hereditary retinal dystrophies, vitreoretinal diseases, myopia or hypermetropia (more than 6 diopters), history of uveitis. After exclusion of poor-quality scans (with a signal strength index < 60), finally 330 eyes (from 168 patients) were taken for final analysis. No child had any signs of diabetic retinopathy on fundus examination and color fundus photography.

We asses factors of diabetes metabolic control: glycated hemoglobin A1c (HbA1c) current to the study, and mean value for whole diabetes duration (minimum 4 tests per year), daily insulin dose per kilogram of weight (DID/kg), mean total daily insulin (taken from pump memory or profile led on hospital, TDI) and dose of insulin at breakfast (as one of markers connected with insulin resistance). The other metabolic factors assessed in the study were thyroid function (thyreothropin (TSH), free thyroxine (fT4), free triiodothyronine (fT3)), lipids (levels of total cholesterol, LDL-cholesterol, HDL-cholesterol, triglycerides (TG)), serum uric acid, plasma creatinine, level of 1.25(OH)D3 vitamin, current and mean microalbuminuria, and creatinine in daily urine collection. Systolic and diastolic pressure was measured on the right arm after a 5 min rest in seated position (mean from 4 measurements was taken for analysis). The following demographic data were reported in the study: weight, height, BMI, BMI z-score, age, age at diabetes onset, diabetes duration.

BMI z-score was reported using LMS method based on Box–Cox transformation [15,16]:

$$z-score\left(x\right)=\frac{{\left(\frac{X}{M}\right)}^{L}-1}{LxS}$$

where *X* is measured anthropometric parameter (ex. height, BMI), *M* is median of the value, *L* is power of Box-Cox’s transformacy, and *S* is variability coefficient. *L*, *M*, and *S* values were taken from reference tables for chosen anthropometric parameter for determined age and sex (were printed by Kułaga et al.) [17,18].

Additionally, we checked the effect of the presence of ketoacidosis at the time of diabetes onset and influence of continuous subcutaneous insulin infusion (CSII) use duration. The MDI group included 81 children (mean diabetes duration 3.38 ± 3 years) treated by pens in scheme basal-bolus-functionally, for which mean dose of insulin analogue was calculated depending on amount of food and correction, or children with stiff doses for main meals. The CSII group consisted of 87 children treated with an insulin pump for more than a year (mean duration of CSII 3.74 ± 1.9 years, mean duration of diabetes 5.47 ± 3.3 years). However, not all the patients strictly followed the recommendations of diabetes care team (stiff doses for meal, not weight meals).

**Figure 1:**
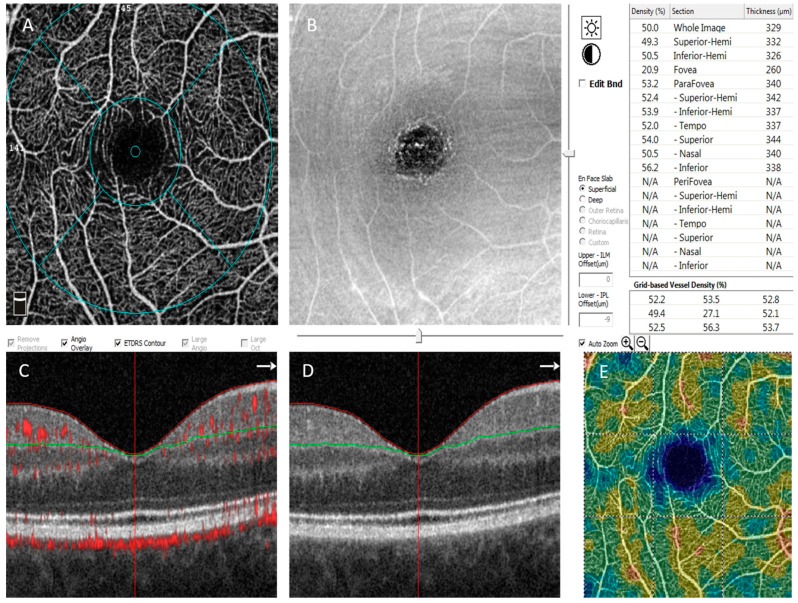
Representative OCTA results of a patient with diabetes (**A**) superficial retinal plexus, (**B**) en-face OCT image, (**C**,**D**) OCT B-scan, (**E**) superficial vessel density map and vessel density results (%) above.

Commercially available RTVue XR Avanti with AngioVue (Optovue, Fremont, CA, USA), with 3 mm × 3 mm images of macula centered on the foveola, was used to perform OCTA. The technique for taking scans was previously described and used in our earlier studies [13,14]. All subjects were dilated with 1% tropicamide eye drops before examination. Each en face OCTA image contains 304 × 304 pixels created from the intersection of the 304 vertical and the 304 horizontal B-scans. The equipment automatically segments the area into four layers, including superficial capillary plexus layer (SP) (Figure 1), deep capillary plexus layer (DP), outer retinal layer and choriocapillaries. The SP en face image was segmental with an inner boundary at 3 µm beneath the internal limiting membrane and outer boundary set at 15 µm, beneath the inner plexiform layer, whereas the deep capillary plexus en face image was segmented with inner boundary 15 µm beneath the inner plexiform layer and an outer boundary at 70 µm beneath the inner plexiform layer. Vessel density is calculated as the percentage of the area occupied by following blood vessels in the selecting region, which enables the quantitative assessment of microvasculature. Whole superficial capillary vessel density (wsVD) and whole deep vessel density (wdVD), as well as foveal thickness (FT)(µm) and parafoveal thickness (PFT) (µm), were analyzed. The data of both eyes of patients were taken into analysis independently, because of possible intra-eye differences and metabolic parameters that influence both eyes.

**Figure 2:**
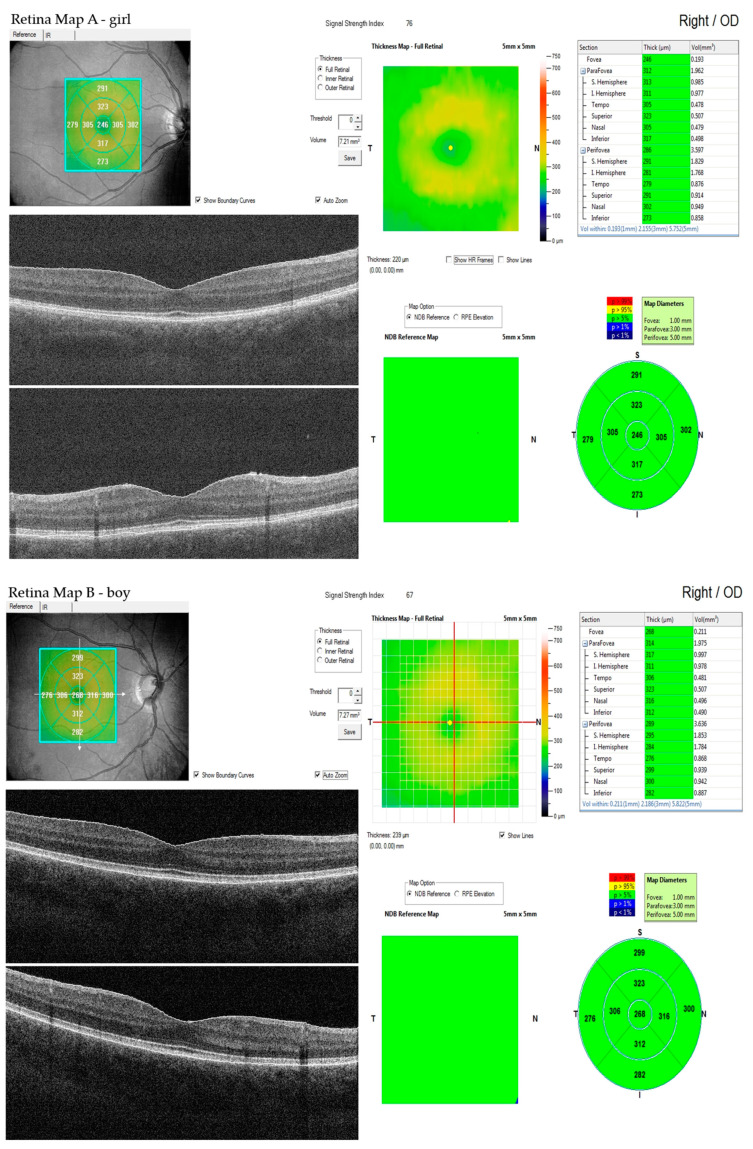
Representative OCT image reflecting measurement and the standard Retinal Thickness Map showing the foveal and parafoveal thicknesses. (**A**) girl, (**B**) boy.

Groups of girls and boys were statistically different in OCT parameters: foveal thickness (FT) (girls 250.4 +/− 17.9 vs. boys 260.2 +/− 19.2, *p* < 0.00001) and parafoveal thickness (PFT) (314.97 +/− 17.1 vs. 321.25 +/− 15.3, *p* < 0.0005), but not in OCTA parameters. (Figure 2A,B).

The study was approved by the Bioethics Committee of Children’s Memorial Health Institute in Warsaw and followed the tenets of the Declaration of Helsinki. A written informed consent was obtained from the patients’ legal guardians and from patients > 16 years old following the explanation of the nature of the non-invasive study.

## 3. Statistical Analysis

Data were described by mean and standard deviation, minimum and maximum values, and median. For the analysis of correlations between parameters, multiple regression analysis was used. The study group was divided dependently of the gender and therefore the influence of all parameters was analyzed for girls and boys separately. The comparison of parameters between girls and boys was performed using the Student’s *t*-test or U Mann–Whitney, depending on normality of distribution. In the first step, all collected descriptive and metabolic data were taken to multiple regression analysis, then for the model, statistically significant parameters were chosen. A level *p* < 0.05 was recognized as statistically significant. Tests were performed using TIBCO Software Inc. (2017) Statistica Version 13 StatSoft Company (Palo Alto, Santa Clara, CA, USA).

## 4. Results

Descriptive data of study population is presented in Table 1. All models are presented in Table 2. In the models in both genders, foveal thickness was dependent on current HbA1c and pH at the onset, and in the group of girls, additionally on diabetes duration, serum creatinine and level of TG; but in boys’ group, on duration of CSII use, levels of fT4 and vitamin D3.

When PFT was analyzed in both genders, only current HbA1c was statistically significant in the models. In the model for girls’ group height, weight, BMI z-score, DID/kg, current microalbuminuria, pH at the onset and level of vit D3 were also statistically significant. However, in the boys’ group, the only addition was the level of fT4 and systolic pressure.

In analysis of parameters influenced by superficial vessel density, only pH at diabetes onset was in both gender models, but in the girls’ group, it was not statistically significant. In the model of wsVD in the girls’ group, the duration of CSII use, systolic pressure, mean HbA1c, and level of uric acid were statistically significant. However, in the boys’ group, the percent of TDI in basal, levels of serum creatinine and total cholesterol were important.

Deep vessel density in both groups depended on the level of LDL fraction of cholesterol, and in boys additionally only on serum creatinine level, but in girls it also depended on CSII use duration, pH at the diabetes onset, TDI, and level of TSH.

## 5. Discussion

We detected significant risk factors set for development of OCT and OCTA results in children with type 1 diabetes, and they were not identical for both genders. It should be highlighted that for all parameters, multiple regression models were statistically significant, despite the lack of differences for most of the OCT parameters between boys and girls. In our previous study, we detected significantly higher central foveal and parafoveal thickness in the group of boys, but it rather confirms the physiological differences between genders, not especially in diabetic children [14]. We should point out that almost all (except the HbA1c) metabolic parameters in both genders were strictly within the norm for healthy population (Table 1). In our studied cohort, in the boys’ group, serum creatinine level, free thyroxine level and systolic pressure were more frequently important, whereas in the girls’ group, parameters of insulin dosage and serum uric acid (Table 2) were more significant. In girls, demographic parameters that more frequently appeared were weight, BMI z-score, and lipids profiles- triglycerides and LDL-cholesterol fraction, which could suggest that elements of metabolic syndrome could play a greater role. In both genders, OCT and OCTA results were frequently influenced by current level of HbA1c, duration of CSII use and pH at the diabetes onset. This is interesting and different from our other studies, where the current level of HbA1c was not such an important factor [13,14]. It is consistent with the study by Romero et al., of patients with T1D with 15 years’ follow up, where gender was not a significant factor for DR in the multivariate logistic regression model, but the most important determinants were arterial hypertension, macroangiopathy, LDL-cholesterol, and higher (>8%) HbA1c [19]. Significant influence of CSII as a treatment method and impact of diabetic ketoacidosis (DKA) were also previously detected in our study, but influence of this last parameter was not so dominant as in this current analysis [18]. Jeziorny et al. compared OCT results of patients with DKA and non-DKA (OCT scans performed at that moment), and did not detect a difference in retinal nerve fiber layer (RNFL) [20]. Salardi et al., in a group of 230 patients with childhood onset of T1D, did not detect differences in retinopathy frequency after 20 years of observation either [21], but of course it is long time, and many other parameters would have influenced the retina and disturbed the results.

In their interventional study, Gallego et al. showed that over long time periods, blood pressure influences DR risk [22]. In our analysis, systolic pressure, but not diastolic pressure, was a significant factor for changes in retinal vascularity. In contrast to this were the results of a study by Harrison et al., where diastolic pressure (DBP) was positively associated with retinal thickness in a group of patients with type 2 diabetes (T2D) non-proliferative diabetic retinopathy (NPDR) [23]. Multiple regression analysis performed by these authors did not reveal associations between increased HbA1c and DBP in patients without retinopathy, but only in patients with retinopathy. They did not observe any associations between systolic and diastolic pressure and any vessel caliber measures in the group with diabetes, but a negative correlation did exist in the healthy control group. A study led by Bek on a small group of patients with diabetic retinopathy, who were tested at 1-to-2-week intervals for up to a year, found no association between the number of retinal microaneurysms, haemorrhages and hard exudates and arterial blood pressure [24]. Głowińska-Olszewska et al. revealed that, in young persons with T1D, patients with retinopathy had significantly higher systolic blood pressure (133 ± 19 mmHg) in comparison to patients without complications (117 ± 14 mmHg), and when compared to healthy controls (115 ± 8 mmHg) [25]. However, we could not exclude the option that this was coincidence, not cause. We observed significant associations, even though our groups have much less pressure, and no retinopathy. The relationship between arterial hypertension and diabetic macular edema could be explained by an increase in hydrostatic pressure, which, in turn, increases perfusion pressure in the vascularity [26]. A review of 15 randomized controlled studies supports the beneficial effect of lowering the blood pressure in prevention of DR for up to 4–5 years [27], and there are suggestions that, in T1D patients, antihypertensive drugs (ACE-inhibitor) could be useful even in the presence of normotension (50% reduction in progression of retinopathy by at least one level, and with the lisinopril therapy for proliferative retinopathy the odds ratio was 0.18) [28].

We observed the influence of the lipids profile (LDL-cholesterol fraction and triglycerides) on deep vessel density, and in the girls’ group on foveal thickness. The associations between serum lipid profiles and DR have been widely studied, but have shown different results, probably due to the variety of methods, the studied groups, and ethnic dissimilarities [29,30,31]. LDL-cholesterol and triglycerides are known to cause endothelial dysfunction via local inflammation and production of advanced glycation endproducts [32].

In both genders, serum uric acid level was a significant factor, but was more frequent in the group of girls. Uric acid, because of its proatherogenic properties—increasing platelet adhesiveness and activation of endothelial cells and platelets—is known as an important risk factor for diabetes vascular complications [33]. Rising serum uric acid levels cause vascular leakage and increased macular thickness. Xia et al. revealed that uric acid might be a risk factor for diabetic retinopathy [34]. However, it must be pointed out that these studies were conducted in the groups of patients with T2D, with a high level of serum uric acid. In our study, patients had a normal value, but with statistically significant difference between boys and girls, which may suggest that even a slight increase in a uric acid level may affect the proper growth and maturity of the retina during puberty. This finding needs further investigation, but recommendation of early treatment in adolescents with T1D should be considered to achieve lower uric acid concentration.

In the group of boys more frequently than in the group of girls, serum creatinine level was an important factor. This factor was also different in those groups, but it is physiologically normal due to different mass of muscles. In a group of Asian T2D patients with DR, Zhang et al. observed significantly higher mean serum creatinine and lower mean eGFR than in age-matched controls [35]. Similarly, Srivastav et al. reported that increased serum urea and creatinine levels were associated with increased severity of DR [36]. Both retina and kidney are organs of low vascular resistance and supplied by small vessels, highly susceptible to fluctuations in blood flow [37].

Increased insulin resistance or hiperinsulinemia in patients with T1D (receiving insulin) could be determined directly only by an insulin clamp procedure, but clinically could be indirectly measured by a parameter of a daily insulin dose per kilogram of weight (DID/kg), total daily insulin (TDI) or insulin dose at breakfast. In our analysis, these parameters exist more frequently in the group of girls, similarly to other characteristic factors for metabolic syndrome (MS) (LDL -C, TG and uric acid), including weight, although this parameter has not been significant in the previous studies [13,14]. The fasting hyperinsulinemia in healthy individuals, independently of the age and arterial hypertension, has a negative impact on the retinal vessel status [38]. Nitric oxide released from endothelial cells is also triggered by insulin, which is an important signal, resulting in vasodilation and reduced vascular resistance, which influences blood pressure [39]. Estimating the prevalence of DR in 2551 participants with the metabolic syndrome, a higher number of MS components increased the risk of DR (adjusted for HbA1c, age, sex, duration of diabetes) [40]. Other studies have found that the presence of hyperinsulinemia and dyslipidemia in type 2 diabetes was associated with the onset of microvascular complications [41,42], similar to our results.

In conclusion, we did not reveal a strict set of risk factors that are different for the different genders in children with T1D, but what is important is that we repeatedly observed, in many parameters of OCT and OCTA, the influence of DKA and current metabolic control, serum creatinine and duration of CSII use are the most important factors for retinal thickness and vessel densities in both genders. For girls, elements of metabolic syndrome (uric acid and triglycerides) and parameters of insulin amount were more pronounced. Notwithstanding, our hypothesis that there are different risk factor sets should be further studied in T1D patients with and without DR, which should allow more pronounced differences to be distinguished. This is the first study of a large group of children with T1D without DR, which analyzes so many factors: demographic, as well as metabolic, biochemic, and management factors, and especially adapted to gender. This is the first time when parameters such as DKA, and TDI or DID/kg in groups of children were analyzed. All clinicians know that metabolic control, DKA prevention, proper dosing of insulin (not excessive) and strict control of other biochemic parameters such as lipids, uric acid or thyroid hormones are essential for preventing late diabetic complications, but randomized controlled trials are needed to confirm our thesis. This was a single site study, and children whose parents wanted to participate were more frequently poorer metabolically controlled, which was our limitation. The most important finding from this study was the observed significant influence of parameters strictly within the norm, such as systolic pressure, uric acid, or lipids, on changes in OCT and OCTA parameters, which could be the target for future intervention studies.

## Figures and Tables

**Table 1:** Characteristic of studied population divided into genders.

Parameter	Girls (Mean and SD)	Boys (Mean and SD)	*p* Value
Diabetes duration (years)	4.96 ± 3.8	4.24 ± 3.6	*p* = 0.07
Age at the diabetes onset (years)	8.09 ± 3.7	8.35 ± 3.8	*p* = 0.5
Age (years)	13.07 ± 3.6	12.6 ± 3.6	*p* = 0.2
Weight (kg)	45.9 ± 16.9	48.8 ± 19.3	*p* = 0.1
Height (cm)	153.3 ± 16.95	157.5 ± 21.5	*p* = 0.06
BMI z-score (kg/m^2^)	−0.1045 ± 1.01	−0.1645 ± 1.1	*p* = 0.6
HbA1c current (%)	8.4 ± 1.9	8.1 ± 1.6	*p* = 0.2
HbA1c mean (%)	8.2 ± 1.4	7.9 ± 1.6	*p* = 0.1
TSH	3.27 ± 6.6	2.34 ± 1.1	*p* = 0.07
fT4	1.05 ± 0.3	1.1 ± 0.48	*p* = 0.3
fT3	2.95 ± 0.64	2.97 ± 0.61	*p* = 0.8
Cholesterol total (mg/dL)	174.1 ± 32.6	160.9 ± 30.1	*p* < 0.0002
LDL cholesterol (mg/dL)	91.33 ± 25.4	81.35 ± 23.6	*p* < 0.0003
HDL cholesterol (mg/dL)	66.53 ± 14.4	63.6 ± 18.06	*p* = 0.1
Triglycerides (mg/dL)	81.35 ± 44.98	78.35 ± 45.6	*p* = 0.6
Uric acid	3.63 ± 0.6	4.18 ± 0.96	*p* < 0.000001
Creatinine plasma	0.57 ± 0.1	0.68 ± 0.2	*p* < 0.000001
1.25(OH)D3 vitamin	24.95 ± 7.95	24.1 ± 7.79	*p* = 0.4
Microalbuminuria current	16.1 ± 25.8	8.9 ± 7.37	*p* < 0.003
Microalbuminuria mean	12.8 ± 19.2	8.1 ± 8.8	*p* < 0.02
Creatinine in daily urine collection	1.94 ± 7.4	5.1 ± 17.26	*p* = 0.08
Systolic pressure (mmHg)	107.7 ± 9.1	110.92 ± 11.9	*p* < 0.02
Diastolic pressure (mmHg)	64.78 ± 8.7	65.95 ± 9.7	*p* = 0.3
Duration of pump use (years)	3.08 ± 1.9	3.45 ± 2.1	*p* = 0.2
Total daily insulin dose (units)	34.59 ± 16.1	36.5 ± 19.9	*p* = 0.4
Daily dose per 1 kg of weight (μ/kg)	0.766 ± 0.25	0.737 ± 0.26	*p* = 0.3
Percent of TDI in basal/100 (%)	0.35 ± 0.12	0.31 ± 0.11	*p* < 0.01
pH at the diabetes onset	7.3167 ± 0.1	7.3036 ± 0.1	*p* = 0.4

**Table 2:** Comparison of multiple regression models for boys and girls for different OCT parameters.

Parameter	Girls	Boys
FT	Model R = 0.38, *p* < 0.00035 HbA1c current β = −2.7, *p* < 0.0005 Serum creatinine β = 25.1, *p* < 0.04 pH at the diabetes onset β = 35.7, *p* < 0.03 Duration of CSII use β = 0.99, *p* = 0.1 Diabetes duration β = −1.01, *p* < 0.02 TG β = 0.08, *p* < 0.05	Model R = 0.36, *p* < 0.001 HbA1c current β = −2.4, *p* < 0.007 Serum creatinine β = −2.8, *p* = 0.7 pH at the diabetes onset β = 33.4, *p* < 0.05 Duration of CSII use β = 2.64, *p* < 0.005 Level of 25(OH)D3 β = 0.46, *p* < 0.02 FT4 β = −5.87, *p* < 0.05
PFT	Model R = 0.61, *p* < 0.00000 HbA1c current β = −2.99, *p* < 0.00003 Microalbuminuria current β = 0.46, *p* < 0.003 pH at the diabetes onset β = 29.85, *p* < 0.03 Level of 25(OH)D3 β = 0.47, *p* < 0.003 BMI z-score β = −13.0, *p* < 0.0000 Weight β = 1.56, *p* < 0.0000 Height β = −1.03, *p* < 0.0000 DID/kg β = −15.48, *p* < 0.003 Serum uric acid β = −4.06, *p* = 0.07	Model R = 0.46, *p* < 0.00001 HbA1c current β = −3.44, *p* < 0.0000 Microalbuminuria current β = 0.37, *p* < 0.03 Systolic pressure β = 0.35, *p* < 0.008 fT4 β = −6.35, *p* < 0.005 pH at the diabetes onset β = 11.64, *p* = 0.3 Level of 25(OH)D3 β = 0.19, *p* = 0.1 Age β = −0.6, *p* = 0.08 BMI z-score β = −1.75, *p* = 0.09
wsVD	Model R = 0.32, *p* < 0.003 HbA1c mean β = −0.35, *p* < 0.01 Systolic pressure β = 0.06, *p* < 0.009 Duration of CSII use β = −0.3, *p* < 0.02 Serum uric acid β = −0.77, *p* < 0.03 pH at the diabetes onset β = 3.24, *p* = 0.1	Model R = 0.35, *p* < 0.001 Serum creatinine β = −3.07, *p* < 0.006 Duration of CSII use β = 0.23, *p* < 0.02 Total cholesterol β = −0.01, *p* < 0.03 Systolic pressure β = −0.02, *p* = 0.1 Serum uric acid β = 0.24, *p* = 0.3 pH at the diabetes onset β = −3.02, *p* = 0.1
wdVD	Model R = 0.44, *p* < 0.00002 LDL cholesterol β = 0.03, *p* < 0.00003 pH at the diabetes onset β = 4.42, *p* < 0.009 TSH β = −0.49, *p* < 0.03 Duration of CSII use β = −0.28, *p* < 0.003 TDI β = 0.02, *p* < 0.04 Serum uric acid β = −0.49, *p* = 0.059 TG β = −0.06, *p* = 0.058	Model R = 0.45, *p* < 0.00003 LDL cholesterol β = −0.02, *p* < 0.0005 Serum creatinine β = −2.67, *p* < 0.005 Height β = 0.02, *p* < 0.05 pH at the diabetes onset β = −1.99, *p* = 0.1 Duration of CSII use β = 0.08, *p* = 0.2 Percent of basal insulin β = 1.33, *p* = 0.2 Microalbuminuria current β = −0.02, *p* = 0.2

## Data Availability

Data available on request due to restrictions.
